# Short-Term Results of Ivabradine *versus* Metoprolol: The Effects on Atrial Fibrillation in Patients Undergoing Off-Pump Coronary Artery Bypass Grafting

**DOI:** 10.21470/1678-9741-2021-0201

**Published:** 2022

**Authors:** Esra Erturk Tekin, Mehmet Ali Yeşiltaş, İsmail Haberal

**Affiliations:** 1 Department of Cardiovascular Surgery, Mersin City Training and Research Hospital, Mersin, Turkey.; 2 Department of Cardiovascular Surgery, Bakirkoy Dr. Sadi Konuk Training and Research Hospital, Istanbul, Turkey.; 3 Department of Cardiovascular Surgery, Istanbul University-Cerrahpasa Institute of Cardiology, Istanbul, Turkey.

**Keywords:** Ivabradine, Metoprolol, Beta-Blocker, Atrial Fibrillation, Coronary Artery Bypass, Erythrocyte Transfusion, Ventricular Fibrilation, Follow-Up Studies

## Abstract

**Introduction:**

Classic coronary artery bypass grafting (CABG) surgery involves diastolic cardiac arrest under cardiopulmonary bypass, while off-pump CABG (OPCABG) has become widespread in recent years.

**Methods:**

174 patients who underwent OPCABG were included in the study. Patients were divided into two groups. Group I (n=90) received ivabradine and Group M (n=84) received metoprolol before surgery until postoperative day 10. Intraoperative arrhythmias and hypotension were recorded. Postoperative atrial fibrillation (AF) and arrhythmia, mortality and morbidity rates were assessed based on the 30-day postoperative follow-up.

**Results:**

There were no significant differences in the intraoperative amount of inotropic support and red blood cell transfusion between groups (P=0.87 and P=0.31). However, the rates of intraoperative arrhythmias and hypotension were not significantly higher in Group M (P=0.317 and P=0.47). Ventricular tachycardia/ventricular fibrillation (VT/VF) was observed in 2 patients in both groups. Postoperative AF occurred in 7 patients (7.7%) in Group I and in 10 patients (11.9%) in Group M. Although there was a trend towards a higher prevalence of AF in Group M patients, this did not reach statistical significance. In addition, mortality and morbidity rates were comparable between groups.

**Table:** Abbreviations, Acronyms & Symbols

AF	= Atrial fibrillation
AV	= Atrioventricular
CABG	= Coronary artery bypass grafting
CI	= Cardiac index
CPB	= Cardiopulmonary bypass
ETCO_2_	= End-tidal carbon dioxide
ICU	= Intensive care unit
IQR	= Interquartile range
LVEF	= Left ventricular ejection fraction
MI	= Myocardial infarction
OPCABG	= Off-pump CABG
PCI	= Percutaneous coronary intervention
VF	= Ventricular fibrillation
VT	= Ventricular tachycardia

## INTRODUCTION

Coronary artery bypass grafting (CABG) surgery is still the main choice for severe coronary artery diseases^[1]^. Classic CABG involves diastolic cardiac arrest under cardiopulmonary bypass (CPB), while off-pump (*i.e.*, on a beating heart) CABG (OPCABG) has become widespread in recent years^[2-4]^. OPCABG is recommended in porcelain aorta, which is a contraindication for CPB, low left ventricular ejection fraction (LVEF), persistent angina, and acute myocardial infarction (MI)^[5,6]^.

As OPCABG involves distal anastomosis in a beating heart, the rate of graft failure is higher than that of on-pump CABG. Additionally, OPCABG requires technical skills and a high level of experience. Therefore, concerns regarding the quality of the anastomosis discourage surgeons from performing OPCABG^[7]^. Cardiac movement at the site of the anastomosis is another concern in OPCABG. However, these movements can be minimized using mechanical stabilizers and drug-induced (pharmacological) bradycardia^[8]^. Although the latter is a theoretically simple method, it may lead to a variety of side effects and even low cardiac output. For pharmacological bradycardia, beta-blockers (β-blockers), ivabradine, and diltiazem can be used. The choice of drug is of utmost importance to ensure maximum benefit and minimum risk^[9,10]^.

Atrial fibrillation (AF) accounts for approximately 30% of all CABG cases. In addition, other arrhythmias can be seen in these patients^[11]^. Regardless of the type of surgery (*i.e.*, on-pump or off-pump), postoperative arrhythmias are associated with increased morbidity and mortality rates^[12]^. Current guidelines recommend the use of β-blockers in the preoperative period, unless contraindicated, to reduce the risk of AF following cardiac surgery^[13]^. Previous studies have also shown that β-blockers decrease morbidity and mortality in chronic heart failure and after cardiac surgeries. These agents have several effects, including reducing myocardial contractility, blood pressure and myocardial oxygen demand, and slowing intracardiac conduction, as well as antiarrhythmic effects. Additionally, these agents exert their effects on the peripheral vascular system and airways, and these effects may vary depending on the agent used and the existing disease and comorbidities of the patient^[14]^.

Ivabradine is the first selective sinus node I(f) channel inhibitor^[15]^. It is indicated for the treatment of patients in sinus rhythm or with a heart rate above the target value receiving β-blockers in optimal dose or in those in the maximally tolerated dose of β-blockers^[16]^. Ivabradine decreases the tendency to spontaneous diastolic depolarization and slows down the heart rate at rest and during exercise^[17]^. However, there are still a limited number of clinical studies investigating the efficacy of ivabradine in the prevention and treatment of postoperative arrhythmias in patients with left ventricular dysfunction or conduction abnormalities undergoing CABG^[18]^. Therefore, in the present study, we aimed to evaluate the short-term effects of preoperative ivabradine *versus* metoprolol on intra- and postoperative AF in patients undergoing OPCABG.

## METHODS

### Study Design and Study Population

This single-center, retrospective study was conducted between January 1^st^, 2018, and February 10^th^, 2020. A total of 642 patients who underwent CABG during the study period were screened. Medical records were retrieved from the hospital database and patient files. Of these patients, 274 underwent OPCABG. The inclusion and exclusion criteria are listed in Table 1. Finally, a total of 174 eligible patients who fulfilled the inclusion criteria were included in the study. All patients were informed about the potential benefits and risks of the procedure, and written informed consent was obtained. The study protocol was approved by the local ethics committee with Approval No. 65355327-604.01.02-E.134-28. The study was conducted in accordance with the principles of the Declaration of Helsinki.

**Table 1:** Inclusion and exclusion criteria.

Inclusion criteria	Exclusion criteria
· Elective CABG	· Emergency CABG
· ≥18 years of age	· Receiving any β-blocker in the preoperative period
· EF >30% or <50%	· ≥3-vessel CABG
· <3-vessel CABG	· <18 age years of age
· CABG on a beating heart	· Conversion from OPCABG to conventional CABG
· Left internal mammary artery harvesting as arterial graft and use of a venous graft	· Intolerance to metoprolol or ivabradine in the preoperative period (<60 bpm)
· Stable angina	· Intolerance to metoprolol or ivabradine in the postoperative period (<60 bpm)
· No electrocardiographic change (*e.g.*, ST-segment elevation, bundle branch block)	· Existing preoperative arrhythmia (AF, atrial flutter, second- or third-degree AV block, VF)
· Not receiving any preoperative antiarrhythmic drug	· EF <30% or >50%
· Completion of preoperative medication	· Use of arterial grafts alone
· Normal sinus rhythm	· Use of venous grafts alone
· Heart rate >60 to <90 bpm	· Renal failure
	· Decompensated heart failure (NYHA class IV)
	· COPD
	· Known hypersensitivity to metoprolol or ivabradine

AF=atrial fibrillation; AV=atrioventricular; CABG=coronary artery bypass grafting; COPD=chronic obstructive pulmonary disease; EF=ejection fraction; NYHA=New York Heart Association; OPCABG=off-pump coronary artery bypass grafting; VF=ventricular fibrillation

Patients were divided into two groups. Group I (n=90) received ivabradine 5 mg/day b.i.d. and Group M (n=84) received metoprolol 50 mg/day b.i.d. five days before surgery until postoperative day 10. Intraoperative arrhythmias and hypotension were recorded. Postoperative AF and arrhythmia, mortality and morbidity rates were assessed based on the 30-day postoperative follow-up.

### Premedication and Anesthesia Protocol

Preoperative preparation included routine monitoring before anesthesia induction in the operating room (*i.e.*, electrocardiography, pulse oximetry, and noninvasive arterial blood pressure). Two peripheral venous accesses were established using 16- and 18-gauge needles, and radial artery cannulation was performed to monitor invasive arterial blood pressure. Following preoxygenation for 3 min, anesthesia induction was initiated by infusion of remifentanil (2.5 µg/kg-^1^) for 4 min and midazolam (0.1 mg/kg-^1^). Rocuronium bromide (0.6 mg/kg) was given as a muscle relaxant.

Following endotracheal intubation, mechanical ventilation was initiated. A nasopharyngeal probe was inserted to monitor the core body temperature, and internal jugular vein cannulation was performed to measure central venous pressure. Mechanical ventilation was provided with an end-tidal carbon dioxide (ETCO_2_) of 35 to 40 mmHg and a tidal volume of 6 mL/kg in all patients. In both groups, maintenance of anesthesia was achieved using infusion of desflurane and remifentanil (0.125 µg/kg-^1^) with an oxygen/air (1 L/1 L) mixture. Dräger Primus® anesthesia workstation (Dräger Medinzintechnik, Lübeck, Germany) was used to monitor the anesthetic gas concentration. Soda lime (Sorbo-Lime®, Berkim Kimya, Turkey) was used as the CO_2_ absorbent. Rocuronium bromide infusion was maintained at 0.3 mg/kg-^1^ every 30 to 45 minutes. In case of increased mean arterial pressure despite increased desflurane concentration and additional remifentanil infusions, nitroglycerin was used. If the mean arterial pressure and heart rate decreased by 20% from baseline, fluid infusion was increased at the same remifentanil infusion dose, and values were monitored every 2 minutes. In case of a persistent decrease, intravenous ephedrine was administered to maintain hemodynamic stability.

### Surgical Procedure

All operations were performed through sternotomy with a midsternal incision. The left internal mammary artery was harvested. Prior to surgery, anticoagulation was maintained with heparin 100 IU/kg to achieve an active coagulation time of >250 seconds. The pericardium was opened widely to the left to facilitate cardiac positioning. To elevate the base of the heart, a single deep pericardial traction suture was placed in two-thirds of the posterior pericardium, from the inferior vena cava to the left inferior pulmonary vein. Special care was taken to avoid any injury to the phrenic nerves, the left lower pulmonary lobe, and the esophagus. Deep sutures and left pericardial sutures were retrieved, and the apex of the heart was elevated to the left for better visualization by the surgeon. The pericardium in the right aspect was incised through the diaphragm to the phrenic nerve and was opened widely to allow the heart to be placed in the pleural space.

The operating table was placed in the right decubitus Trendelenburg position to facilitate visualization of the target arteries in the lateral and inferior aspects of the heart. At our center, we routinely use a novel cardiac positioning system that allows lifting the heart securely, rather than pushing the apex of the heart, positioning easily, and accessing the target vessels with preserved functional geometry of the heart (Maquet ACROBAT stabilizer and XPOSE positioner; Maquet GMBH & Co, Rastatt, Germany). During surgery, cardiac positioning devices were placed on the left apex to visualize the lateral wall and left circumflex coronary artery branches; on the apex to visualize the anterior wall of the heart (left anterior descending artery) and inferior wall (posterior descending artery); and on the acute margin of the heart to uncover the right coronary artery.

A sterile, humidified CO_2_ insufflator was used to maintain a clean surgical field. Heparin was administered, and after a short waiting time, arteriotomy was performed. Following the arteriotomy procedure, an intracoronary shunt (Medtronic Inc., MN, USA) was inserted into the coronary artery depending on the coronary artery diameter. Anastomosis was performed using interrupted 7-0 Prolene sutures. The intracoronary shunt was removed from the coronary artery just prior to the completion of anastomosis. All distal anastomoses were then completed, and proximal anastomoses were sutured onto the aorta using a side-biting clamp and 6-0 Prolene sutures. During side-clamping, special care was paid to avoid plaque formation, and the sites were gently washed with diluted heparin solution. The number and duration of coronary vascular anastomoses and the total operation duration were recorded. Following surgery, all patients were transferred to the intensive care unit (ICU) with intubation.

### Clinical and Laboratory Assessment

Baseline clinical and laboratory findings were documented for preoperative risk assessment. The grafts used during surgery, the operation duration, the amount of blood products used, intraoperative arrhythmias, and hypotension requiring inotropic support were recorded. In the postoperative period, time to extubation, stroke, and amount of inotropic support and blood products were also evaluated. During the intra- and postoperative periods, ST-T changes were examined for postoperative MI, and all patients were monitored for arrhythmias. In the postoperative period, laboratory tests, including complete blood count, troponin I, creatine kinase-myocardial band, and arterial blood gas (lactate, oxygen, and CO_2_), were recorded and compared between groups.

Postoperative AF was defined as an episode of AF that occurred during hospitalization after OPCABG and was not reversible with electrolyte supplementation or oxygenation. Renal failure was defined as a glomerular filtration rate of <90 mL/min/1.73 m^2^. Third-degree atrioventricular (AV) block was an indication for pacing, and these patients underwent pacemaker implantation.

### Outcome Measures

The primary outcome measure was 30-day mortality. Secondary outcome measures included intra- and postoperative arrhythmias during the 30-day follow-up, such as AF, ventricular fibrillation (VF) and ventricular tachycardia (VT); need for pacing; bradycardia (<60 bpm); and usual morbidities, such as stroke and chronic renal failure.

### Statistical Analysis

The Statistical Package for the Social Sciences version 21.0 for Windows (IBM Corp., Armonk, NY, USA) software package was used for data evaluation and analysis. Categorical variables are presented as frequencies (n) and percentages (%), and numerical variables are presented as mean ± standard deviation or median value (interquartile range [IQR]). Normality distribution of data was analyzed using the Kolmogorov-Smirnov test. The chi-square test and Fisher’s exact test were used to compare the differences in proportions of categorical variables between groups. Independent-samples t-tests and Mann-Whitney U tests were used for the comparison of continuous variables between two independent groups. A *P*-value <0.05 was accepted as statistically significant.

## RESULTS

Of a total of 174 patients, 90 were included in Group I, and 84 were included in Group M. There were no statistically significant differences in OPCABG risk factors, such as age, sex, diabetes, smoking, body mass index, and chronic obstructive pulmonary disease, between groups. Mean preoperative LVEF was 39.37±3.40% in Group I and 38.9±4.9% in Group M, indicating no significant difference (*P*=0.461). The mean baseline heart rate was 79.5±9.2 bpm in Group I and 80±9.3 bpm in Group M, indicating no significant difference (*P*=0.722). Six patients in Group I and five patients and Group M had previous percutaneous coronary intervention (PCI) (*P*=0.847). Baseline demographic and clinical characteristics of the patient groups are shown in Table 2.

**Table 2:** Baseline demographic and clinical characteristics of the patient groups.

Variable	Ivabradine (n=90)	Metoprolol (n=84)	*P*-value
Age (years)	63.6±9.6	64.1±11.3	0.753*
Sex (male), % (n)	73.3% (n=66)	76.2% (n=64)	0.665^†^
BMI (kg/m2)	28.1±5.2	27.9±5.0	0.796*
Diabetes, % (n)	34.4% (n=31)	39.2% (n=34)	0.411^†^
Hypertension, % (n)	44.4% (n=40)	50% (n=42)	0.463^†^
Current smoker, % (n)	16.6% (n=15)	15.4% (n=13)	0.831^†^
Dyslipidemia, % (n)	23.3% (n=21)	21.4% (n=18)	0.763^†^
Baseline HR	79.5±9.2	80±9.3	0.722*
LVEF (%)	39.37±3.40	38.9±4.9	0.461*
Previous myocardial infarction, % (n)	4.44% (n=4)	3.57% (n=3)	1^‡^
Previous PCI, % (n)	6.66% (n=6)	5.95% (n=5)	0.847^†^
Renal impairment, % (n)	4.44% (n=4)	4.76% (n=4)	1^‡^
COPD/asthma, % (n)	5.55% (n=5)	5.95% (n=5)	0.911^†^
Systolic blood pressure (mmHg)	129.9±16.0	130.7±15.5	0.738*
Diastolic blood pressure (mmHg)	76.1±9.9	77.0±9.7	0.546*

* Independent samples t-test;

† chi-square test;

‡ Fisher’s exact test. Data are given as mean ± standard deviation or number and frequency, unless otherwise stated. *P*<0.05 indicates statistical significance. BMI=body mass index; COPD=chronic obstructive pulmonary disease; HR=heart rate; LVEF=left ventricular ejection fraction; PCI=percutaneous coronary intervention"

Furthermore, the mean heart rate was significantly lower during distal anastomosis in Group I than in Group M (*P*<0.001). In Group M, an increased heart rate endangered the quality of anastomosis in seven patients (7.77%); therefore, intravenous metoprolol at a dose of 3.19 was given. However, none of the patients in Group I needed an additional dose.

Pacemakers were needed in 6 patients (6.66%) in Group I and in 3 patients (3.57%) in Group M. In Group I, one of the six patients who needed pacing underwent permanent pacemaker implantation during the follow-up. The need for pacing was abolished in the other three patients on postoperative day 1 and in the remaining two patients on postoperative day 2. In Group M, the three patients requiring pacemaker implantation after surgery no longer needed it as of postoperative day 1. Although there was a trend towards a higher prevalence of the need for a pacemaker in Group I patients, this did not reach statistical significance (Table 3).

**Table 3:** Intra- and postoperative data.

Variable	Ivabradine (n=90)	Metoprolol (n=84)	*P*-value
Number of grafts	1.62±0.58	1.7±0.66	0.49^§^
Operation time (min)	154.1±30.2	158.2±35.5	0.412*
Intraoperative arrhythmia	3 (3.33%)	6 (7.1%)	0.317^‡^
Intraoperative hypotension	5 (5.55%)	7 (8.33%)	0.470^†^
Need for inotropic agent	9 (10%)	9 (10.7%)	0.877^†^
Intraoperative HR	60.3±7.11	79.8±11.5	<0.001*
Blood transfusion	1.7±1.4	1.6±1.3	0.31^§^
Need for pacemaker implantation	6 (6.66%)	3 (3.57%)	0.499^‡^
Postoperative AF	7 (7.7%)	10 (11.9%)	0.360^†^
Postoperative VT/VF	2 (2.22%)	2 (2.38%)	1^‡^

* Independent samples t-test;

† chi-square test;

‡ Fisher’s exact test;

§ Mann-Whitney U test. "Data are given as mean ± standard deviation or number and frequency, unless otherwise stated. *P*<0.05 indicates statistical significance. AF=atrial fibrillation; HR=heart rate; VF=ventricular fibrillation; VT=ventricular tachycardia"

There was no significant difference in length of hospital and ICU stay between groups (*P*=0.28 and *P*=0.52, respectively). In addition, mortality and morbidity rates were comparable between groups (*P*=0.49 for both). Two patients in each group developed low cardiac output, leading to mortality (Table 4).

**Table 4:** Postoperative morbidity and mortality.

Variable	Ivabradine (n=90)	Metoprolol (n=84)	*P*-value
Length of ICU stay (median [min-max]) (day)	1 (1-4)	1.1±1.6	0.52^§^
Intubation time (median [min-max]) (h)	6 (4-13)	6(3-14)	0.36^§^
Length of hospital stay (median [min-max]) (day)	5(4-12)	6(4-12)	0.28^§^
Stroke	2 (2.22%)	2 (2.38%)	1^‡^
Mortality (%)	2 (2.22%)	2 (2.38%)	1^‡^

§ Mann-Whitney U test;

‡ Fisher’s exact test. Data are given as mean ± standard deviation or number and frequency, unless otherwise stated. *P*<0.05 indicates statistical significance. ICU=intensive care unit

## DISCUSSION

Approximately 30% of patients undergoing CABG develop postoperative AF. In addition to AF, arrhythmias can also be seen in these patients^[11]^. Regardless of the type of surgery (*i.e.*, on-pump or off-pump), postoperative arrhythmias are associated with increased morbidity and mortality rates^[12]^. Patients with postoperative AF are more vulnerable to other complications, including intraoperative MI, congestive heart failure, respiratory failure, prolonged hospital stay and increased healthcare costs^[19]^. Postoperative AF has a variable degree of effects from transient complications without serious consequences to severe acute renal injury, hemodynamic instability, heart failure, stroke, and even death^[20]^.

Meta-analyses and systematic reviews investigating the prevention of postoperative arrhythmias have shown that medical treatment modalities, including β-blockers, sotalol and amiodarone, are effective in the prevention and/or treatment of postoperative AF^[21,22]^. The use of β-blockers is difficult and still controversial in patients with conduction abnormalities, severe

There were no significant differences in the mean number of grafts and the mean operation duration between groups (*P*=0.49 and *P*=0.40, respectively). In addition, no significant differences in the intraoperative amount of inotropic support and red blood cell transfusion were observed between groups (*P*=0.87 and *P*=0.31, respectively). However, the rates of intraoperative arrhythmias and hypotension were not significantly higher in Group M (*P*=0.317 and *P*=0.47, respectively). VT/VF was observed in 2 patients in both groups. Postoperative AF occurred in 7 patients (7.7%) in Group I and in 10 patients (11.9%) in Group M. Although there was a trend towards a higher prevalence of AF in Group M patients, this did not reach statistical significance.

Six of the seven patients in Group I returned to normal sinus rhythm with antiarrhythmic drugs, while the other patient returned to normal sinus rhythm following cardioversion. Normal sinus rhythm was restored with antiarrhythmic drugs in five patients and following cardioversion in two patients in Group M. The three remaining patients in Group M were discharged with AF without signs of hemodynamic instability. Two of these patients returned to normal sinus rhythm during follow-up, while the other patient was followed up with AF in the outpatient setting. The intra- and postoperative data of the patients are presented in Table 3.

left ventricular dysfunction, or active bronchospasm; therefore, other antiarrhythmics must be used for patients undergoing cardiac surgery. Ivabradine, the first selective sinus node I(f) channel inhibitor, slows the heartbeat without affecting myocardial contractility and has protective effects on low LVEF^[23]^. It has been shown to be effective in the treatment of stable coronary artery disease, left ventricular systolic dysfunction, and chronic heart failure^[24]^.

OPCABG has morbidity and mortality rates comparable to those of conventional CABG in high-risk patients^[4-7]^. In a review including 16,900 patients, there were no significant differences in morbidity and mortality rates between patients undergoing OPCABG and those undergoing conventional CABG; however, OPCABG exerted a more protective effect against infection and renal dysfunction^[25]^. OPCABG is recommended in porcelain aorta, which is a contraindication for CPB, LVEF, persistent angina, and acute MI^[5,6]^. However, challenges of the technique are debated, including epicardial instability during distal anastomosis due to the nature of the distal anastomosis site. Mechanical stabilizers are used to avoid epicardial instability and to maintain local stabilization. The use of mechanical stabilizers has been shown to be associated with a significant increase in anastomotic patency rates^[26-28]^. However, these devices cannot always be actively utilized or may remain limited in certain settings. In such cases, pharmacological bradycardia offers technical simplicity during anastomosis in OPCABG.

Currently, several medical agents are available to induce pharmacological bradycardia, including β-blockers (*i.e.*, esmolol, metoprolol tartrate, metoprolol succinate, propranolol, atenolol, and nadolol), diltiazem, verapamil, digoxin, amiodarone, flecainide, ibutilide, and ivabradine^[29]^. Theoretically, these agents may also be helpful in reducing cardiac displacement. In clinical practice, β-blockers are commonly used in the treatment of cardiovascular disease. In general, CABG is required in patients with previous MI and impaired LVEF; therefore, medications that reduce cardiac oxygen demand and prevent intra- and postoperative arrhythmias are of vital importance. In addition, the agent to be used must be free from deleterious effects, without affecting myocardial contractility, blood pressure, and ventricular repolarization. Of note, medications with potential respiratory side effects, such as β-blockers, must be avoided following OPCABG^[30]^. Thus, medications with lower arrhythmia burden and higher surgical comfort during OPCABG should be selected.

In a study, Iliuta et al.^[18]^ divided cardiac surgery patients into three groups as follows: metoprolol 100 mg/day, n=176; metoprolol 50 mg/day + ivabradine 5 mg b.i.d., n=179; and ivabradine 5 mg b.i.d., n=172. All patients were followed for postoperative AF and arrhythmias for 30 days. The rate of third-degree AV block and pacing was significantly higher in the metoprolol alone group (12.5%), while this rate was the lowest in the ivabradine alone group (2.91%) (*P*<0.0001). However, postoperative AF and arrhythmias during hospitalization were less frequently seen in the group receiving the combined treatment (metoprolol + ivabradine) than in the monotherapy groups (*P*<0.001). The associated relative risk indicated a higher protective value against postoperative AF with the combined treatment than with metoprolol alone (-2.9 *vs.* -1.8). Based on the 30-day follow-up outcomes, ivabradine monotherapy showed superiority over metoprolol monotherapy. In our study, the rate of postoperative AF was not significantly lower in Group I (n=7, 7.7%) than in Group M (n=10, 11.9%) (*P*=0.36). However, there was not a significantly higher number of patients in Group I who needed pacing in our study (*P*=0.49). This can be attributed to the lower LVEF of the patient population included in our study. In addition, we administered premedication for five days before surgery, while Iliuta et al.^[18]^ used premedication for only two days.

In a multicenter study, Koester et al.^[31]^ investigated the efficacy of additional ivabradine 5 mg/day b.i.d. to β-blocker in patients with stable angina. Patients were followed for four months, and, at the end of follow-up, the mean heart rate decreased from 84.3±14.6 bpm to 72.0±9.9 bpm (*P*<0.001). None of the patients developed bradycardia, and the treatment was well tolerated in more than 90% of patients. In another study, Werdan et al.^[32]^ examined the addition of ivabradine (5 mg/day b.i.d.) in patients with chronic stable angina despite the use of β-blockers following PCI and assessed symptoms and heart rhythm at one and four months. Mean heart rate was 83.1±11 bpm at baseline, 69.4±8.8 bpm at one month, and 64.4±7.6 bpm at four months (*P*<0.0001). Throughout the study, angina episodes were seen less frequently in these patients (*P*<0.0001). Consistent with these findings, in our study, the mean heart rate was 79.5±9.2 bpm at baseline, while it decreased to 60.3±7.11 bpm during OPCABG in Group I. This decline was significant compared to baseline values and that of Group M (*P*<0.001). As such, a decline in heart rate prolongs the diastolic phase and increases the coronary artery supply; it is associated with a lower risk of new-onset MI during OPCABG. In addition, this decline ensures comfort for the surgical team during surgery.

In their study, Nguyen et al.^[10]^ compared the efficacy of ivabradine *versus* placebo in patients who experienced low cardiac output and who were prescribed dobutamine following elective CABG. In the ivabradine arm, the medication was given as infusion therapy, and the median heart rate, which increased due to the effects of dobutamine, decreased from 112 bpm (range, 105 to 120) to 86 bpm (range, 78 to 96) (*P*<0.001). In the placebo arm, the median heart rate decreased from 112 bpm (range, 104 to 120) to 104 bpm (range, 89 to 118), indicating a statistically significant difference between groups (*P*<0.05). Furthermore, the median systolic blood pressure was 110 mmHg (range, 93 to 118), the median cardiac output was 4.7 L/min-^1^ (range, 3.6 to 5.4), and the median cardiac index (CI) was 2.5 L/min-^1^ (range, 2.0 to 2.8) at baseline in the intravenous ivabradine arm; after treatment, these values were 125 mmHg (range, 114 to 139), 5.3 L/min-^1^ (range, 4.5 to 6.5), and 2.9 L/min-^1^ (range, 2.4 to 3.4), respectively (*P*<0.05 for all). Likewise, in our study, preoperative oral ivabradine yielded a similar decrease in heart rate of patients with baseline LVEF. Although cardiac output and CI were not examined in our study, lower intraoperative inotropic support was achieved with ivabradine.

In a multicenter, double-blind, randomized, placebo-controlled study, Fox et al.^[33]^ examined the effect of additional ivabradine on standard therapy in patients with stable coronary artery disease without clinical heart failure. Nearly 20,000 patients from 1,139 centers in 51 countries were screened. Patients were divided into two groups: ivabradine group (n=9.539) and placebo group (n=9,544). Adverse events, including second- and third-degree AV block, AF, bradycardia, and supraventricular tachycardia during the study were evaluated. Asymptomatic bradycardia developed in 1,718 patients (18.0%) in the ivabradine arm and in 223 patients (2.3%) in the placebo arm (*P*<0.001). Second-degree AV block was seen in 44 patients (0.5%) and 31 patients (0.3%) in the ivabradine and placebo arms, respectively (*P*=0.13). Third-degree AV block was detected in 20 patients (0.2%) and 19 patients (0.2%) in the ivabradine and placebo arms, respectively (*P*=0.87). In addition, 508 patients (5.3%) in the ivabradine arm and 362 patients (3.8%) in the placebo arm developed AF (*P*<0.001). A total of 137 patients (1.4%) in the ivabradine arm and 65 patients (0.7%) in the placebo arm developed supraventricular tachyarrhythmia (*P*=0.13). The rates of bradycardia and AF were significantly higher in patients treated with ivabradine. In our study comparing ivabradine *versus* metoprolol, we also did not find a significantly higher rate of bradycardia or a need for pacing in the ivabradine group. Six patients (6.6%) in the ivabradine group and three patients (3.57%) in the metoprolol group required pacing (*P*=0.49). Additionally, the rate of postoperative AF was not significantly lower in Group I (n=7, 7.7%) than in Group M (n=10, 11.9%) (*P*=0.36). Two patients in each group developed VF and VT (*P*=1). The higher rate of bradycardia in the ivabradine group is consistent with the results of Fox et al.^[33]^

These findings suggest that ivabradine may have a more prominent effect on heart rate, although it has no activity at the AV node and does not alter the ventricular rate in AF. In the present study, we monitored the patients for AF in the ICU setting and applied immediate interventions for possible causes of AF, such as electrolyte imbalance and reduced oxygenation, which may have yielded different results from the aforementioned study. In addition, Fox et al.^[33]^ administered ivabradine 10 mg/day b.i.d. in their study, which can explain the discrepancy between the results.

In their study, Abdel-Salam and Nammas^[9]^ compared the efficacy of preoperative ivabradine, bisoprolol, or combination therapy for the prevention of postoperative AF in patients undergoing CABG. Patients were divided into two groups as follows: ivabradine (48 h preoperatively, then 1 week postoperatively) 5 mg b.i.d. for 24 h, then 7.5 mg b.i.d. thereafter in patients who could tolerate it (Group 1, n=212); bisoprolol 5 mg b.i.d. (Group 2, n=288) or combination therapy (ivabradine as before + bisoprolol 5 mg once daily) (Group 3, n=240). The primary endpoint was the incidence of AF at the 30-day follow-up. There was no significant difference in baseline LVEF or clinical characteristics among the groups. However, the incidence of AF at the 30-day follow-up was 15.1% (n=32) in the ivabradine group, 12.2% (n=35) in the bisoprolol group, and 4.2% (n=10) in the combination therapy group (*P*<0.001). Subgroup analysis showed a significantly lower incidence of AF in the first two days in the combination therapy group (*P*<0.01). In our study, the rate of postoperative AF was not significantly lower in Group I (n=7, 7.7%) than in Group M (n=10, 11.9%) (*P*=0.36). The difference between the results can be attributed to the fact that the number of patients with valve diseases was higher in the ivabradine group in the study of Abdel-Salam and Nammas^[9]^, although without statistical significance. Additionally, prolonged aortic cross-clamp and cardiopulmonary bypass times during CABG may have increased the incidence of AF in patients treated with ivabradine.

### Limitations of the study

There are some limitations to this study. Its retrospective design and relatively small sample size are the main limitations. In addition, no comparison was made between OPCABG and conventional CABG. Most patients undergoing CABG have coronary artery disease, and those receiving antiarrhythmic drugs were not included in the study. Further large-scale, prospective, randomized controlled studies including these patient groups are warranted.

## CONCLUSION

As a result, in our study, ivabradine did not reduce the risk of AF in OPCABG patients compared to metoprolol. However, by effectively reducing the heart rate, surgical comfort during the operation was greatly improved. Ivabradine appears to be a useful choice to provide a more comfortable and effective anastomosis during OPCABG. It may be an effective strategy to reduce heart rate in selected OPCABG patients who cannot be given the targeted beta-blocker dose.
